# Cytokine storm and severe hepatitis in pregnancy due to herpes simplex virus 2

**DOI:** 10.1007/s15010-023-02092-x

**Published:** 2023-09-27

**Authors:** Alessandra Mularoni, Andrea Cona, Lùcia Ribeiro Dias, Matteo Bulati, Rosalia Busà, Salvatore Castelbuono, Davide Lo Porto, Giada Pietrosi, Rosa Liotta, Pier Giulio Conaldi, Paolo Antonio Grossi, Mario Luppi

**Affiliations:** 1 Unit of Infectious Diseases, ISMETT-IRCCS Istituto Mediterraneo per i Trapianti e Terapie ad Alta Specializzazione, Via Tricomi 5, 90127 Palermo, Italy; 2 Infectious Diseases Department, Centro Hospitalar Universitário São João, Porto, Portugal; 3 Department of Research, ISMETT-IRCCS Istituto Mediterraneo per i Trapianti e Terapie ad Alta Specializzazione, Palermo, Italy; 4 Department for the Treatment and Study of Abdominal Disease and Abdominal Transplantation, ISMETT-IRCCS Istituto Mediterraneo per i Trapianti e Terapie ad Alta Specializzazione, Palermo, Italy; 5 Pathology Unit, Department of Diagnostic and Therapeutic Services, ISMETT-IRCCS Istituto Mediterraneo per i Trapianti e Terapie ad Alta Specializzazione, Palermo, Italy; 6 Infectious and Tropical Diseases Unit, Department of Medicine and Surgery, University of Insubria-ASST-Sette Laghi, Varese, Italy; 7 Section of Hematology, Department of Medical and Surgical Sciences, University of Modena and Reggio Emilia, AOU Modena, 41124 Modena, Italy

**Keywords:** Pregnancy, Hepatitis, Herpes simplex 2, Viral sepsis, Systemic inflammatory response, Cytokine storm

## Abstract

**Case presentation:**

A pregnant woman developed hepatitis due to a herpes simplex virus 2 primary infection with a severe systemic inflammatory response. Treatment with acyclovir and human immunoglobulin was given and both mother and baby survived.

**Purpose:**

We provide the first description of the inflammatory response associated with herpetic hepatitis in pregnancy.

## Introduction

Herpes simplex virus (HSV) hepatitis is an uncommon disease primarily observed in immunocompromised individuals and pregnant women frequently leading to adverse outcomes. It represents a challenging diagnosis due to its non-specific clinical manifestations, such as fever, gastrointestinal symptoms, and flu-like symptoms. Highly characteristic herpetic lesions are found in less than half of cases [1].

The high mortality observed in HSV hepatitis in pregnancy, up to 39% [1], is mainly due to delayed diagnosis and treatment [2]. Furthermore, the high mortality may be partially attributed to an inadequate systemic inflammatory response, as evidenced in cases of hemophagocytic lymphohistiocytosis (HLH) and macrophage activation syndrome (MAS) triggered by HSV [3–5]. This hypothesis aligns with observations of hyper-inflammatory host responses leading to cytokine storms seen in other viral infections, such as SARS-CoV-2, Epstein–Barr virus (EBV), and human herpes virus 8 (HHV-8) infection [6–8]. A cytokine storm is characterized by uncontrolled systemic hyper-inflammation caused by an excess of cytokines, which correlates with disease severity [9].

We present an unprecedented evaluation of the inflammatory response in a pregnant woman with HSV-induced hepatitis following HSV-2 primary infection, successfully treated with acyclovir and immunoglobulin. We support the inclusion of HSV infection among the pathologic drivers of cytokine storm [9].

## Case

A 41-year-old primigravid woman, at 18 weeks gestation, with acute liver failure was referred to our transplant center for a trans-jugular liver biopsy and assessment for a potential liver transplant. Past medical history was unremarkable. The patient exhibited a ten-day history of persistent fever, headache, and acute hepatitis. Despite outpatient treatment with amoxicillin, cefixime, and acetaminophen, up to 1 gr three times per day for seven days, her symptoms did not improve. At admission (day zero), she was febrile (T 38.7 °C), alert, oriented, and hemodynamically stable. Physical examination revealed severe asthenia, pallor, sub-icteric sclera, and abdominal pain. Laboratory findings showed anemia (hemoglobin 8.3 g/dL), lymphopenia (1.4 × 10^3^/μL), elevated transaminases (AST 7864 units/L, ALT 3012 units/L), hypoalbuminemia (1.5 g/dL), INR 1.4, increase in total bilirubin (1.6 mg/dL), and creatinine was 0.57 mg/dl. Inflammatory markers were elevated, with C-reactive protein (CRP) at 136 mg/L, (normal value 0–5 mg/L) procalcitonin (PCT) at 10.3 ng/mL, and ferritin at 36,185 ng/mL. Minimal peri-hepatic ascites was observed on abdominal ultrasound. Empiric antibiotic treatment with meropenem was initiated. The pathologist, at first evaluation of urgent liver biopsy, observed cytolytic liver damage with extensive centrilobular necrosis (acinar zone 3), suggestive of drug-induced damage. On day one after admission, the peripheral blood smear showed activated reactive and apoptotic lymphocytes. Serological tests for hepatitis A virus (HAV), hepatitis B virus (HBV), hepatitis C virus (HCV), cytomegalovirus (CMV), toxoplasmosis, and hepatitis E virus (HEV) were negative for acute infection. The plasma viral load of HSV-1 and 2, CMV, adenovirus, and varicella-zoster virus (VZV) was negative. Fecal samples tested negative for adenovirus, and molecular testing for respiratory viruses was also negative.

**Fig. 1:**
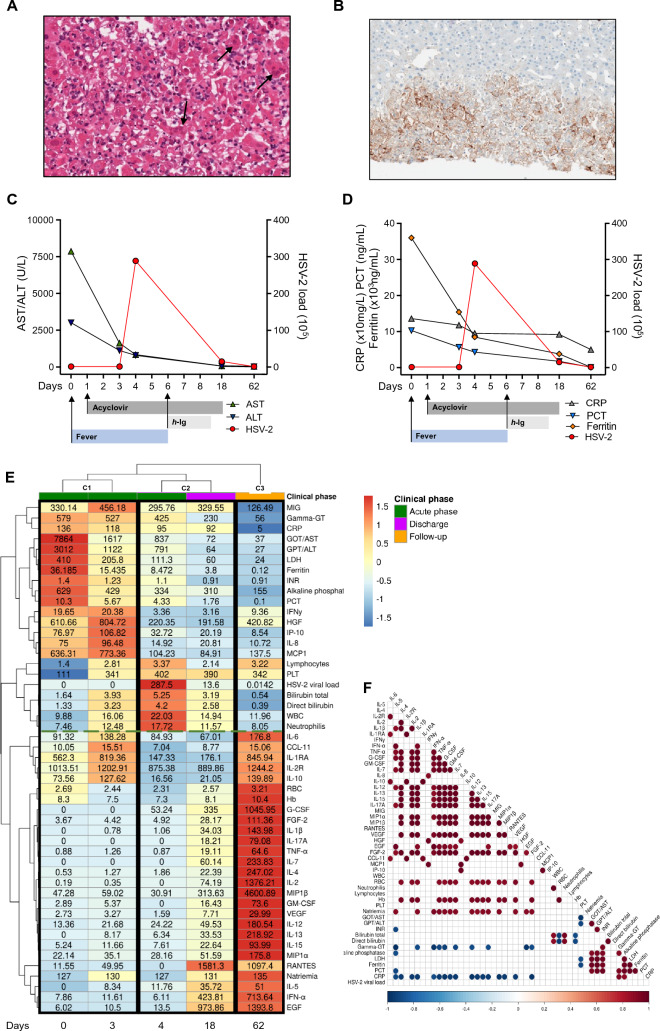
**A** hematoxylin and eosin stain of liver biopsy. Arrows demonstrate HSV inclusions with nuclear molding, and multinucleation, consistent with Cowdry-type inclusions (H&E 20x). **B** immunohistochemical stain strongly positive for HSV-2 (H&E 10x). **C** Transaminases (AST-ALT) levels and HSV-2 viral load, repeated twice, at five designated time points (day: 0–3–4–18–62). **D** levels of inflammatory markers (C-reactive protein, procalcitonin, and ferritin) and HSV-2 viral load in all time points. **E** the clustered heatmap shows the levels of 30 circulation cytokines/chemokines and 19 clinical biomarkers (rows) detected on different days (columns), normalized by z-score transformation. The analysis reveals three discernible temporal clusters corresponding to C1 (day 0 and day 4 since admission), C2 (day 4 since admission, and day 18, corresponding to discharge), and C3 (day 62, follow-up time). The green dotted line represents the two major biomarkers clusters. **F** pairwise Pearson correlation analyses were conducted to assess the associations between cytokine expression levels and laboratory values across the five-time points. Red: positive correlation; Blue: negative correlation. Only correlations exceeding the 95% confidence interval were considered statistically significant. *ALT* alanine transaminase, *AST* aspartate transaminase, *CRP* C-reactive protein, *EGF* epidermal growth factor, *FGF* fibroblast growth factor, *G-CSF* granulocyte colony-stimulating factor, *gamma-GT* gamma-glutamyl transferase, *GM-CSF* granulocyte–macrophage colony-stimulating factor, *Hb* hemoglobin, *HGF* hepatocyte growth factor, *HSV-2* herpes simplex virus 2, *IFN* interferon, *IL* interleukin, *INR* international normalized rate, *IP-10* interferon-gamma inducible protein-10, *LDH* lactate dehydrogenase, *MCP* monocyte chemoattractant protein, *MIG* monokine induced by interferon-gamma, *MIP* macrophage inflammatory protein, *PCT* procalcitonin, *PLT* platelets, *RBC* red blood count, *TNF* tumor necrosis factor, *VEGF* vascular endothelial growth factor, *WBC* white blood count

Despite a negative HSV-1 and -2 viral load, serological analysis demonstrated positive HSV-1/2 IgM and borderline IgG antibodies. Consequently, a histological review of the liver biopsy was requested, revealing numerous cells with viral nuclear inclusions (Fig. 1A) and a highly suggestive morphology for herpes virus cytopathic effects. Immuno-histochemical staining and real-time polymerase chain reaction (PCR) for HSV-2 were positive in the hepatic tissue (Fig. 1B), confirming the diagnosis. This was a primary HSV infection rather than a reactivation, as confirmed by the fourfold rise in IgG title observed four weeks after the first serological evaluation.

Acyclovir treatment was initiated, leading to a progressive reduction in transaminases and inflammatory markers (Fig. 1C and D). However, on the fourth day, the patient’s clinical condition deteriorated, concomitantly with the development of anasarca attributed to severe hypoalbuminemia (1.8 g/dL) and an elevation in total bilirubin level (5.25 mg/dL). On the following day, the HSV-2 viral load became detectable, quantified at 28.750.000 copies/mL. The observed increase in inflammatory markers and high serum ferritin, despite optimized HSV-2 treatment, raised concerns of unregulated hyper-inflammation suggestive of a cytokine storm. Probability of hemophagocytic lymphohistiocytosis (HLH) using H score was 25–40% on day 4 and 80–88% on day 5 [10, 11]. By considering that steroids are usually not recommended in acute HSV infections and that cyclosporin may be unsafe during pregnancy [12].

We decided to administer human polyvalent immunoglobulin at a dose of 400 mg/kg/day for five days (Fig. 1C and D) [13]. The patient started to recover with a reduction in H score (with probability of HLH 25–40% on day 7, 8, 10, and 16–25% on day 12), inflammatory markers and in HSV-2 viral load to 1.361.205 copies/mL within one week. The patient became afebrile while HSV-1/2 DNA on the vaginal swab remained detectable. Thus, we recommended a cesarean delivery.

After a total hospital stay of 20 days, including 19 days of acyclovir treatment, the patient was discharged with normalized inflammatory markers and recovery of liver function. On the follow-up visit, a month after discharge, she was asymptomatic with a viral load of HSV-2 of 120 copies/mL and a detectable HSV-1/2 vaginal swab. Obstetric examination revealed no discernible abnormalities in the fetus. The patient underwent a cesarean delivery at 33+s3 weeks of pregnancy. The newborn was a healthy girl with a birth weight of 1800 gr. HSV-1/2 DNA in plasma and cerebrospinal fluid of the newborn was undetectable. Both mother and baby are alive and well at the 6-month follow-up.

Using Luminex technology and the R software tool (version 4.1.2), we retrospectively analyzed plasma cytokine levels (stored at − 80 °C until their use) at five designated time points (day: 0–3–4–18–62) and correlated them with the clinical data after a z-score transformation. The heatmap generated in Fig. 1E revealed three discernible temporal biomarker clusters. The first cluster, represented by the acute phase (day 0 and day 3 after admission) in the absence of viremia, revealed an impaired specific antiviral immune response and, conversely, the production of cytokines and chemokines, including monokine induced by interferon-gamma (MIG), interferon-gamma (IFNγ), hepatocyte growth factor (HGF), monocyte chemoattractant protein-1 (MCP-1), interferon-gamma induced protein-10 (IP-10), C–C motif chemokine ligand 11 (CCL-11), and interleukin-8 (IL-8), IL-6, IL-1RA, IL-2R, and IL-10, all involved in inflammation. This profile was accompanied by increased hepatic (AST, ALT, bilirubin, gamma-GT, alkaline phosphatase, INR) and inflammatory (CRP, PCT, LDH, ferritin) biomarkers. In the second cluster, the viremic phase (day 4–18), we observed a reduction in the aforementioned inflammatory profile and an increase in total bilirubin, WBC, and neutrophils. Finally, in the third cluster (day 62, follow-up), we observed a complete recovery of hepatic markers and an increase in different cytokines involved in antiviral immunity, suggesting the onset of T cell responses involved in viral clearance and recovery from the infection. In Fig. 1F, we show the pairwise Pearson correlation analysis, which associates cytokine expression levels and laboratory values across the five-time points.

## Discussion and conclusions

We present the first comprehensive description of the inflammatory response observed in a pregnant woman with herpetic hepatitis. The unique immunological landscape of pregnancy, characterized by T cell deficiency, renders pregnant women particularly susceptible to virus-related complications [14]. After a systematic literature search in Pubmed and Web of Science databases to identify cases of hepatitis attributed to HSV-2 published from January 2013 onwards, we identified only thirteen cases, two of which were associated with HLH and fetal death [3, 4]. The occurrence of a cytokine storm was not reported in the remaining patients. In recent years, a growing body of evidence has contributed to our evolving understanding of the systemic inflammatory responses triggered by various infectious agents. Notably, viruses like coronaviruses, influenza, HHV-8 [7], and more recently, SARS-CoV-2 [6] have been associated with hyper-inflammatory host responses, often culminating in cytokine storms. Interestingly, different viral infections may also be associated with distinct patterns of cytokine elevation, emphasizing the intricacies and nuances of the host-virus interplay in immune dysregulation [9]. Despite appropriate treatment, our patient experienced a sudden increase in inflammatory markers concomitant with a deterioration in overall clinical status consistent with the occurrence of a cytokine storm [9], as demonstrated by the circulating levels of different cytokines. In the acute phase (cluster 1), we observed an increase in MIG, IFNγ, HGF, MCP-1, IP-10, CCL-11, IL-8, IL-6, IL-1RA, IL-2R, and IL-10, which have previously been associated with cytokine storms in the context of COVID-19 [6, 8]. Precise thresholds defining elevated cytokine levels are not firmly established and the precise identity of cytokines driving hyper-inflammation in the context of viral-associated cytokine storms remains unknown. We initiated immunomodulatory therapy with human immunoglobulins (day 6) in an attempt to further mitigate the excessive inflammatory response observed, in addition to the antiviral therapy with acyclovir that had been administered immediately upon admission (day 1). The combined therapy probably prevented the organ failure typically associated with a cytokine storm and, on day 18, the patient recovered fully and was discharged with a noticeable decrease in inflammatory markers. The management was challenging due to an exaggerated inflammatory response that prompted us to question if the patient needed an immunomodulatory/immunosuppressive therapy to modulate the cytokine storm. We decided to administer immunoglobulins for their immunomodulatory activity [13]. At follow-up, the patient presented a cytokine profile characteristic of an active antiviral response. Recently, a cytokine storm-like syndrome has been reported in a mice model of ocular HSV primary infection and resulted to be related to a deficiency of M1 macrophages [15]. During pregnancy, the increased risk for certain types of infection indicates important qualitative immunological changes in both innate and adaptive branches [16]. Functional loss of T and B cells has been stated [16], and the characteristic immunomodulatory microenvironment in pregnant women induces a shift toward M2 macrophages phenotype [17]. It could be assumed the susceptibility to hyperinflammation during HSV-2 primary infection in pregnant women is due either to an imbalance in the M1/M2 ratio, or, alternatively, to the impaired ability to mount a cytotoxic or humoral response in the early stage of infection. Conversely, the immune response against virus reactivation differs in terms of speed, strength, and mechanisms involved. Reactivation benefits from the presence of memory immune cells, leading to a faster and more targeted defense. This conceptual framework holds the potential to enhance the clinical management of pregnant women with viral sepsis triggered by HSV infections, providing a rationale for incorporating immunomodulatory approaches in conjunction with acyclovir. Early recognition of HSV sepsis and a related cytokine storm are imperative since HSV-directed treatment is potentially life-saving, as already reported for EBV and HHV-8 [7, 9].

## Data Availability

Data are available from the corresponding author, AC, upon reasonable request.
